# Neglected and non-consented care during childbirth in public health facilities in Central Tigray, Ethiopia

**DOI:** 10.1186/s12884-022-04662-7

**Published:** 2022-05-03

**Authors:** Elsa Tesfa Berhe, Hailay Abrha Gesesew, Paul R. Ward, Teferi Gebru Gebremeskel

**Affiliations:** 1 Department of Reproductive Health, College of Health Sciences, Aksum University, Aksum, Ethiopia; 2 Present Address: Discipline of Public Health, Flinders University, Adelaide, Australia; 3 Department of Epidemiology, College of Health Sciences, Mekele University, Mekele, Ethiopia; 4 Present Address: Centre for Research On Health Policy, Torrens University, Adelaide, South Australia Australia

**Keywords:** Neglected, Non-consented care, Childbirth, Ethiopia

## Abstract

**Background:**

The present study aimed to assess the magnitude and factors associated with neglected and non-consented care during childbirth in public health facilities in Central Tigray, Ethiopia.

**Methods:**

A health facility-based cross-sectional survey supplemented by a qualitative study was conducted from April to May 2020 among women giving birth. We included 415 participants and recruited via a systematic random sampling technique. To collect the data, a pre-tested, face-to-face exit interview using an interviewer-administered structured questionnaire was used. Neglected and non-consented care and its outcomes (yes and no) were the dependent variables, and Socio-demographic data such as (age, educational level, region, and income), and other variables associated with compassionate and respective maternity care were the independent variables. We applied bivariate and multivariate logistic regression to determine predictors for non-consented and non-confidential care components of disrespect or abuse. The in-depth interviews were analyzed using content analysis.

**Results:**

Among the participants, 82.4% and 78.6% had neglected care and non-consented care among women giving birth respectively. No formal education level (AOR: 0.37, 95%, CI (0.18–0.78)) and primary education level (AOR: 0.18, 95%, CI (0.05–0.57))., mode of delivery (AOR 3.79, 95% CI 1.42–10.09), sex of skilled healthcare providers (AOR: 0.56, 95%, CI (0.34–0.93)), number of deliveries in a health Centre (AOR: 1.89, 95% CI (1.03–3.47)) predicted non-consented care, and history ANC (AOR: 8.10, 95% CI (1.33–49.51)), and federal government employee (AOR: 0.24, 95% CI (0.07–0.78)) predicted neglected care during childbirth. In-depth interview result shows the mode of delivery and sex of healthcare providers were factor associated with non-consented care and women's stay at health facilities were factor associated with neglected care.

**Conclusion:**

The level of neglected and non-consented care during delivery was high reflecting substantial mistreatment. Educational level, mode of delivery, sex of skilled healthcare providers, and the number of deliveries in a health Centre were associated with non-consented care, and history ANC and Federal Government employees were associated with neglected care during childbirth. These findings imply the urgent needs or intervention including strengthening of awareness of both patients and healthcare providers on patients' rights and responsibilities and training service providers in patient-centered care and interpersonal communication and relationships to minimize mistreatment.

## Background

The World Health Organization (WHO) defines compassionate and respectful maternity care as the right of all women to receive the highest standard of health care containing: the right to dignity, compassionate, and respectful health services throughout pregnancy, childbirth, and postnatal period [1]. Compassionate and respectful care is not only morally and financially essential, but it is required in many countries through national legislation and/or national health policy [2].

The distribution of compassionate and respectful care of “women” during childbirth has not been well documented [3]. Reports of disrespect and abusive treatment of women giving birth are shared among families and friends in medical settings, and consequences of this to families can be devastating and prevent the mother from accessing health care services delivery in health facilities [4]. In low- and middle-income countries, 19% to 28% of women experience disrespectful and/or abusive treatment from healthcare providers in Health care facilities when giving childbirth [5].

Previous studies in Ethiopia have shown a regional variation in disrespect and abuse. It ranges from 37.5% in Bale Zone [6], to 67.1% in Bahridar [7], and 74.8% in Western Ethiopia [8]. Moreover, qualitative studies in northern Ethiopia also found that disrespect and abuse was a serious obstacle to the utilization of maternal health care [6, 9–11]. The main reasons disrespect and abuse include lack of compassionate and respectful care including negative attitude by the staff, lack of payment satisfaction of health care providers, lack of educational promotion opportunity, weak health system, lack of training, work overload, poor legal implementation, and substandard facilities [12].

The nature, forms, cause, and magnitude of disrespect and abuse of Ethiopian women giving birth is also not documented although reported to have been the cause of maternal mortality and morbidity [13]. However, it has received less attention than other barriers to maternity care during labor and childbirth [13].

To bring maternal voice into maternal health quality and respect care, this study aimed; (i) to evaluate the quality of maternal care and respect from the perspective of consumers and; (ii) to develop the concept of respectful maternal care from the perspectives of clients.

## Methods

### Study design and setting

We have performed a health facility-based cross-sectional survey supplemented by a qualitative study from April and May 2020. The study was conducted in Aksum University Comprehensive and Specialized Hospital (AKUCSH), Saint Marries general hospital, and Adwa general hospital in Central Tigray regional state, northern Ethiopia.

Aksum is the capital of the central zone of Tigray which is located 1024 km north of Addis Ababa and 247 km from Mekele, the capital city of Tigray. According to the plan and finance office of Aksum town (personal communication, 2019), the total population size is 77,723 of which 38,402 are males and 39,321 are females. Axum city has five kebeles, one referral and teaching hospital, one general hospital, two health centers, four health posts, and 10 different level private clinics.

AKUCSH started as a referral hospital in 2014 for serving an estimated 3.6 million population in its catchment areas. Currently, the hospital has around 760 health workers including 19 specialists. The hospital provides emergency, in-patient, and outpatient services at different departments. Currently, the Gynecology & Obstetrics (GYN/OBS) department has two gynecologists /obstetricians, 12 GPs, and 49 midwives. The hospital has 177 beds in four wards: medical, pediatrics, surgical, gynecology, and obstetrics wards. Of these, the maternity ward has 42 beds and delivery and labor wards have 13 beds.

Saint Marries general hospital, the second study setting, was established in 1961 and has 368 health care workers including 17 general practitioners (GPs). In the GYN/OBS department, there are one gynecologist /obstetrician, one Integrated Emergency Surgeon and Obstetrician (IESO), and 18 Midwives. The GYN/OBS department has 28 beds and delivers similar services to AUCSH.

Adwa general hospital, the third study setting, the estimated population of Adwa is 70,440 with females accounting for 50.9% in 2019. It is located 1013 km away from Addis Ababa, the capital city of Ethiopia, and 230 km from Mekele capital city of Tigray regional state. Currently, the hospital has 345 health care workers including 12 general practitioners (GPs). In the GYN/OBS department, there are one gynecologist /obstetrician, one Integrated Emergency Surgeon and Obstetrician (IESO), and 23 Midwives. The GYN/OBS department has 19 beds and delivers and labor wards have 10 beds.

### Study population and sampling process

All immediate (up to 72 h after birth) postnatal mothers who give birth in the selected public hospitals during the study period were included in this study. We excluded immediate postnatal Mothers who were seriously ill or unable to communicate at the time of data collection and who had delivered by cesarean- section.

The sample size was determined using the single population proportion formula. The following parameters were used to calculate the sample size: the proportion of people with disrespect and abuse during women giving birth = 43% [14], 95% CI (**Ζ**_1-α/2_) = 1.96), and 5% degree of marginal error (d). Assuming a 10% non-response rate, the minimum required sample size was 415. The calculated sample sizes (415) were proportionally allocated to each selected hospital based on the client flow which was obtained by referring client registration logbooks. By considering the total 648 frequency of delivery on the two governmental general hospitals and one referral hospital from delivery registration during a month (by the user of the highest and lowest report in the six months then use, 2019 six month report). A systematic random sampling technique was used to collect data using women's delivery registration numbers from the delivery logbook. Data were collected from every 2^nd^ woman who gave birth during the study period at each selected hospital.

We have selected 11 participants for in-depth interviews purposively but interviewed nine. Because of data saturation, i.e. the point where no more significant information was obtained was reached after approximately nine interviews. Each interview was continued until saturation had been reached. The interviews stopped when the narrative became repetitive and no new data were revealed.

### Variables, measurement, and data collection process

The dependent outcome variables for the quantitative study are non-consented care and neglected care. Non-consented care was measured using seven indicators. These indicators were as follows: (a)The health professionals introduce themselves and greets the mother and her support person, (b) the health professional encourages the mother to ask questions, (c) the health professionals respond to the mother's question with politeness and truthfulness, (d) the health professionals explains what is being done and what to expect throughout labor and birth, (e) the health professionals gives periodic updates on the progress of your labor, (f) The health professional permits the mother to choose the position for birth, and (g) The health professionals obtained consent or permission. All the questions were categorized as (“Yes” and “No”). The respondent’s experience of mistreatment was indicated by two categories: ‟ Non-consented care’’ for women who answer no to at was least one of the criteria and ‟ Consented care ‟women who answer yes for all of the criteria [15].

Neglected care was measured using four indicators. These indicators were as follows: (a) Health care providers ignored you when you called for help, (b) left unattended during the second stage of labor, (c) demonstrating the caring culturally inappropriate way, and (d) the separate mother from the baby without medical indication. All the questions were categorized as (“Yes”, and “No”). The respondent’s experience of mistreatment was indicated by two categories: ‟ Neglected care’’ for women who answer yes to at was least one of the criteria and ‟ Non-neglected care’’ women who answer no for all of the criteria [16].

Federal Government Employee is an officer or an individual; 1. appointed in the civil service by one of the following actions in an official capacity- the. President; a Member or Members of Congress, or the Congress; a member of a uniformed. 2. engaged in the performance of a Federal function under the authority of law or an Executive act; and 3. subject to the supervision of an individual named by paragraph (1) of this subsection while engaged in the performance of the duties of his position [17].

Delivery service payment: Types and amounts of user fees for maternity services including card fees (registration fee required before consultation), consultation fees, charges for delivery services, lab services, and essential commodities for maternity service, such as drugs and supplies and payment before a woman can receive treatment, including treatment for obstetric emergencies [18].

Availability of instrument: Availability of basic supplies like suture, linens, blanket, delivery instrument [19].

The independent variables in the study included Socio-demographic data such as (age, sex, educational level, region, income), and other variables associated with compassionate and respective maternity care.

The in-depth interview focused on understanding the underlying neglected care and non-consented care of women giving birth by the health professionals.

The tool used to measure neglected and non-consented care during childbirth was developed using a two-phased approach. First, a comprehensive literature review was conducted on neglected and non-consented care during childbirth. From the literature, specific indicators were selected that were reviewed by experts with vast experience in the field of maternal health. The refined set of indicators was prepared in English and then translated into the local language (Tigrigna) and back into English by a professional person. To establish face validity and translation quality the questionnaire was tested on 5% of the total sample size determined for this study participant outside of the study site by data collectors and supervisors during training. A few questions, language clarity, and information were revised and the questionnaire was finalized for the study. In total, 11 indicators were finally retained based on neglected and non-consented care behavior defined by Bowser and Hill [15, 20].

Two trained nurses collected the data, and another trained nurse supervised the data collection process supervisors recruited and trained for two days. The supervisors supervised the process of data collection, checked the completeness and consistency of data.

The in-depth interview focused on understanding the underlying reasons neglected care and non-consented care of women giving birth by health professionals. The following probing points were included in the in-depth interview guide containing the following probing questions: (a) could you please describe your experience during childbirth at the facility? Please explain to me what happened Probes; labor history (when and how it started, travel to the facility, admission procedures, waiting time, management before delivery, management during delivery and after delivery), (b) describe the most notable event during the stay in the health facility during the last childbirth?, (c) please narrate to me your experience of friendly and sensitive treatment during your last childbirth?, (d) can you guess the reason for this disrespect and abuse?, (e) What was your response while you were experiencing disrespect and abuse?, (f) What do you recommend be done to decrease disrespect and abuse?, and do you have any information that you went to add?. The interview process was field notes were recorded.

### Data analyses

After being coded, data were entered into the Epi-data software version3.1 and exported to SPSS version 22.00 for analysis. Bivariate logistic regression was done to check the crude association between the factors and outcome variables, and variables with *p*-value < 0.25 were selected for multiple logistic regression analysis to identify independent predictors. Adjusted odds ratio and 95% of CI were calculated to check the strength of association with *P*-values less than 0.05 considered as a significant association. Model fitness was checked through the Hosmer and Lemeshow test. The qualitative component was analyzed using content analysis. Tape-recorded interviews were transcribed and then translated into English by two professionals.

## Results

### Socio-demographic characteristics of study participants

A total of 415 study participants were included in this study making a response rate of 100%. The mean age of the participants was 27.4(SD =  ± 5.1) years, ranging between 17 and 45. Nearly half the participants 205(49.4%) were in the age group 25–34 years. The majority of participants 400(96.4%) were orthodox Christian followers. Nearly half of the participants completed secondary education but 30(7.2%) had no formal education. Nearly 95% of participants did not pay for delivery service. Most of the participants 258(62%) reported that there was full availability of instruments in the hospital.

The in-depth interviews were conducted among nine participants aged between 23 and 40 years old: four of whom were from Adwa general hospital, three from Aksum Saint Marry general hospital, and two from Aksum University comprehensive specialized referral hospital. Table 1 describes the socio-demographic characteristics of the study participants in the quantitative study.

**Table 1:** Socio-demographic characteristics of study participants, Central Tigray, Ethiopia 2020 (*n* = 415)

Category		Frequency	Percent %
Women age	< 25	164	39.5
	25–34	205	49.4
	≥ 35	46	11.1
Ethnicity	Tigray	413	99.5
	Others	2	0.5
Women marital status	Married	402	96.9
	Single	10	2.4
	Divorced	3	0.7
Women religion	Muslim	15	3.6
	Orthodox	410	96.4
Residence	Urban	333	80.2
	Rural	82	19.8
Women education	Collage and above	99	23.9
	Secondary education	206	49.6
	Primary education	80	19.3
	No formal education	30	7.2
Women occupation	Housewife	196	47.2
	Governmental employee	95	22.9
	Merchant	59	14.2
	Farmer	41	9.9
	Student	18	4.3
	Private employer	6	1.5
Husband’s occupation	Merchant	124	29.9
	Daily labor	101	24.3
	Private employer	76	18.3
	Farmer	76	18.3
	Federal Governmental employee	38	9.2
Delivery service payment	No	391	94.2
	Yes	24	5.8
Ability to pay	No	83	20
	Yes	332	80
Availability of instrument	No	157	37.8
	Yes	258	62.2

Delivery service payment: Types and amounts of user fees for maternity services including card fees (registration fee required before consultation), consultation fees, and charges for delivery services, lab services, and essential commodities for maternity service, such as drugs and supplies and payment before a woman can receive treatment, including treatment for obstetric emergencies

Ability to pay: Ability to pay means the ability of women to pay for the cost of services

Availability of instrument: Availability of instrument: Availability of basic supplies like suture, linens, blanket, and delivery instrument

### Obstetric history of mother

Four hundred and nine (98.6%) participants had ANC follow-up for the last-born child. Nearly half the participants 190 (45.8%), had received ANC service at the public general hospitals followed by 146(35.2%) at public health centers. Nearly three-quarters 291(70.1%) of the women had more than four ANC visits to the health facility. One in three women (28.7%) gave birth more than twice at the health institution. Table 2 describes the detailed obstetric characteristic of the study participants.

**Table 2:** Obstetric characteristics of the study participants, Central Tigray, Ethiopia, 2020 (*n* = 415)

Category		Frequency	Percent%
ANC follow up	No	6	1.4
	Yes	409	98.6
ANC provider	Midwife	325	78.5
	Doctor	45	10.8
	Nurse	10	2.4
	I don’t know	29	7
Place of receiving ANC	Government’s general hospital	190	45.8
	Government’s health center	146	35.2
	Government’s referral hospital	29	7.0
	Non-Government's health institution	44	10.6
Number of ANC follow up	≥ four	291	70.1
	< four	118	28.4
Parity	One	143	34.5
	Two-four	232	55.9
	≥ Five	40	9.6
Number of previous deliveries in an HF	< Two	296	71.3
	≥ Two	119	28.7
HCP conducting delivery	Midwifery	379	91.3
	Doctor	15	3.6
	Nurse	2	0.5
	She did not know the HCP	19	4.6
Sex of HCP conducting delivery	Male	207	49.9
	Female	208	50.1
Mode of recent delivery	normal delivery	247	59.5
	Episiotomy or normal tear	142	34.2
	vacuum extraction or forceps delivery	26	6.3
Stayed at HF after delivery	Yes	326	78.6
	No	89	21.4
Number of days stayed at HF	One-two days	57	64
	> two days	32	36
Complication during delivery	No	342	82.4
	Yes	73	17.6

*ANC* Antenatal Care, *HCP* Health Care Provider, *HF* Health facility

Complication during delivery: (perennial tears, abnormal heart rate of the baby; water breaking early; perinatal asphyxia; shoulder dystocia; excessive bleeding, prolonged labor, retained placenta, malpresentation.etc.)

### Neglected and non-consented care during facility-based childbirth

Of the 415 participants, 342(82.4%) reported having experienced at least one form of neglected care during facility-based childbirth while the remaining 73(17.6%)) did not experience any form of neglected care and 326(78.6%) reported having experienced at least one form of non-consented care during facility-based childbirth while the remaining 89(21.4%) did not experience any form of non-consented care. Table 3 presents the type of neglected and non-consented care during facility-based childbirth. This was supported by the in-depth interview findings.

**Table 3:** Type of neglected and non-consented care during facility-based childbirth in Central Tigray, north Ethiopia**,** 2020 (*n* = 415)

Category and types of neglected and non-consented care	Experience D and A	
	**Yes %**	**No%**
**Non—consent to care**	**326 (78.6)**	**89 ( 21.4)**
The health care worker introduces themselves and greets the mother and her support person	43 (10.4)	372 (89.6)
The health care worker encouraged the mother to ask questions	151 (36.4)	264 (63.6)
The health care worker responds to the mother's question with politeness and truthiness	261 (62.9)	154 (37.1)
The health care worker explains what is being done and what to expect throughout labor and birth	280 (67.5)	135 (32.5)
The health care worker gives periodic updates on the progress of your labor	292 (70.4)	123 (29.6)
Health care workers permit the mother to the choice of position for birth	164 (39.5)	251 (60.5)
A health care worker can obtain consent or permission	111 (26.7)	304 (73.3)
**Neglected care**	**342 (82.4)**	**73 (17.6)**
Health care workers ignored you when you called for help	55 (13.3)	360 (86.7)
Left unattended during the second stage of labor	38 (9.2)	377 (90.8)
Demonstrating the caring culturally inappropriate way	13 (3.1)	402 (96.9)
The separate mother from the baby without medical indication	314 (75.7)	101 (24.3)

Experiences during childbirth could leave lasting negative impressions in the minds of the women. And regretted having birth in a health facility due to neglected and non-consented care they received in the facilities.

For example, 25 years gravid 2 Para one said *“When I cried and I shout because of aggravating labor pain one female health professional criticized and shout at me, and left me alone. When I called her to know why she did, no response".*

Moreover, 40 years old mother from a Rural area also said *"I came to this hospital from the health center Rahya for better diagnosis and the health care worker told me that the labor condition was at an early stage. After a few minutes my labor pain starts pushing down and I called the health care worker but the health care worker ignored me. After I gave up hope, in the waiting room,* the baby was born *with the help of my uncle and sister. After birth, the health care worker asked me to help, but I refused because I was angry and crying. My baby has in critical condition because of distress (shortness of breathing) and admittance to the neonatal intensive care unit (NICU)…*I believe my baby changed from normal breathing to *distress (shortness of breathing)* due to the provider ignoring my call which delayed a timely *diagnosis*…”.

In the same way, a 25-year-old woman said *"At midnight I was admitted and did not diagnose immediately, but after 2 h check me. The health care provider later simply brought a pair of scissors, which was terrifying, and cut me… During repair and suture of the tear and laceration around my vagina pinched and slapped me, after little pose, I decide never to come to this hospital again."* Whilst talking about her experiences, this woman has a sad face and looked distressed.

### Factors associated with non-consented care

Age of participant, residence, educational level of the women, parity, mode of recent deliveries, number of previous deliveries in an HF, sex of health professionals conducting delivery, facing complication during delivery, and ability to pay for delivery service had a *P*-value of < 0.25 were candidate variables into the multivariate logistic regression.

In multivariate logistic regression analysis, educational level of the women, mode of recent deliveries, Sex of HCP conducting delivery, and the number of deliveries in a health Centre were statically significant associated with non-consented care.

The odds of reporting non-consented care were lower among women with no formal education and primary education level compared to those women with college and above educational level (AOR: 0.37, 95%, CI (0.18–0.78)) and (AOR: 0.18, 95%, CI (0.05–0.57)). Women who had an episiotomy or tear during delivery were four times (AOR 3.79, 95% CI 1.42–10.09) more likely to non-consented care compared to women who had an instrumental delivery.

The odds of non-consented care among women who had only delivered in a health facility once were two times those of women who had delivered in a health facility twice or more(AOR: 1.89, 95% CI (1.03–3.47))). Moreover, the odds of non-consented care were lower when the healthcare provider was male than when the healthcare provider was female (AOR: 0.56, 95%, CI (0.34–0.93)) (Table 4).

**Table 4:** logistic regression analysis of non-consented care and neglected care of its explanatory variables among women giving birth in Central Tigray, north Ethiopia, 2020 (*n* = 415)

**Variable**	**Non-consented care**				**Neglected care**			
	**Yes, n (%)**	**No, n (%)**	**COR (95%CI)**	**AOR (95%CI)**	**Yes, n (%)**	**No, n (%)**	**COR (95%CI)**	**AOR (95%CI)**
Age of participant								
< 25	133 (81.1)	31 (18.9)	1.88 (0.89–3.93)	0.95 (0.34–2.67)				
25–34	161 (78.5)	44 (21.5)	1.60 (0.79–3.26)	0.98 (0.42–2.33)				
≥ 35	32 (69.6)	14 (30.4)	1	1				
Women occupation								
Housewife					166 (82.2)	36 (17.8)	1	1
Merchant					46 (78)	13 (22)	0.6 (0.29–1.25)	0.94 (0.39–2.23)
Farmer					35 (85.4)	6 (14.6)	0.46 (0.19–1.12)	1.08 (0.33–3.58)
Private employer					11 (61.1)	7 (38.9)	0.76 (0.26–2.23)	1.09 (0.33–3.63)
Gov. employer					11 (11.6)	84 (88.4)	0.02 (0.00–0.64)	0.24 (0.1–0.78)^a^
Mode of recent delivery								
Normal delivery	188 (76.1)	59 (23.9)	1.69 (0.71–3.99)	2.75 (0.98–7.70)				
Episiotomy or tear	121 (85.2)	21 (14.8)	3.05 (1.20–7.74)	3.79 (1.42–10.09)^b^				
Instrumental delivery	17 (65.4)	9 (34.6)	1	1				
Sex of health professionals conducting delivery								
Male	153 (79.3)	54 (26.1)	0.57 (0.36–0.92)	0.56 (0.34–0.93)^a^				
Female	173 (83.2)	35 (16.8)	1	1				
Parity								
≥ Five	26 (65.0)	14 (35.0)	1	1				
Two-four	182 (78.4)	50 (21.6)	1.96 (0.95–4.03)	1.06 (0.22–2.56)				
One	118 (82.5)	25 (17.5)	2.54 (1.16–5.54)	0.91 (0.29–2.92)				
Do you have the ability to pay								
Yes	266 (80.1)	66 (19.9)	1.55 (0.89–2.68)	1.08 (0,56–2.09)				
No	60 (72.3)	23 (27.7)	1	1				
Facing complications during delivery								
No complication	274 (80.1)	78 (19.9)	1	1	52 (71.2)	21 (28.8)	0.44 (0.24–0.79)	0.59 (0.26–1.32)
Complication	52 (71.2)	21 (28.8)	0.70 (0.35–1.09)	0.78(0.36–1.39)	290 (84.8)	52 (15.2)	1	1
Women educational								
College and above	88 (88.9)	11 (11.1)	1	**1**	24 (80)	6 (20)	0.55 (0.19–1.62)	0.63 (0.16–2.50)
Secondary education	159 (77.2)	47 (22.8)	0.42 (0.21–0.86)	0.48 (0.19–1.20)	61 (76.3)	19 (23.7)	0.44 (0.20–98)	0.40 (0.15–1.11)
Primary education	61 (76.3)	19 (23.7)	0.40 (0.18–0.90)	0.18 (0.05–0.57)^b^	170 (82.5)	36 (17.5)	0.65(0.32–1.32)	0.56 (0.24–1.30)
No formal education	18 (60)	12 (40)	0.19 (0.07–0.49)	0.37 (0.18–0.78)^b^	87 (87.9)	12 (12.1)	1	1
Experience of delivery in an HF								
< two	242(81.8)	54 (18.2)	1.87 (1.14–3.06)	1.89(1.03–3.47)^a^				
> = two	84 (70.6)	35 (29.4)	1	1				
Residence								
Urban	266 (79.9)	67 (20.1)	1.46(0.83–2.54)	0.87(0.42–1.84)				
Rural	60 (73.2)	22 (26.8)	1	1				
Did you stay in HF after delivery								
Yes					64 (71.9)	25 (28.1)	0.44 (0.25–0.77)	1.77 (0.85–3.69)
No					278(85.3)	48(14.7)	1	1
Do you think there is enough availability of the instrument								
Yes					204 (79.1)	54 (20.9)	1	1
No					138 (87.9)	19 (12.1)	1.92 (1.09–3.39)	1.63 (0.89–2.99)
Did you receive ANC for this child								
Yes					336 (83.1)	65 (16.9)	1	**1**
No					6 (42.8)	8 (57.2)	6.89 (1.77–54.9)	**8.1 (1.33–49.5)**^**a**^
Husband occupation								
Gov. employer					32 (84.2)	6 (15.8)	0.79 (0.28–2.49)	1.83 (1.25–2.78)
Private employer					60 (78.9)	16 (21.1)	0.55 (0.25–0.84)	0.64 (0.17–0.99)
Merchant					107 (86.3)	17 (13.7	0.93 (0.43–2.02)	0.97 (0.38–2.47)
Farmer					69 (87.1)	7 (9.2)	1.46 (0.55–3.85)	3.28 (0.94–11.4)
Daily labor					88 (87.1)	13(12.9)	1	1

*1* Reference, *COR* Crud’s Odd’s ratio, *AOR* Adjusted odds ratio, *CI* Confidence interval

^a^ Statistically Significant variables in bivariate analysis

^b^ Statistically Significant variables in multivariate significance

Data from the in-depth interviews show that women giving birth at health facilities complained of negative experiences of non-consented care due to episiotomy or tear during delivery. Women who had an episiotomy or tear during delivery complained of experience of non-consented care, for example, one woman said "due to episiotomy or tear the main reason the health care provider simply brought a pair of scissors, which was terrifying, and cut me. During repair and suture of the tear and laceration around my vagina pinched and slapped me, after a little pose, I decide never to come to this hospital again”.

### Factors associated with neglected care

In multivariate logistic regression analysis, women’s occupation and receiving ANC for the recent child were statically significant associated with neglected care.

The odds of neglected care among women who did not receive ANC for the recent child were 8 times (AOR: 8.10, 95% CI (1.33–49.51))) more likely as compared to those women who receive ANC for the recent child. Moreover, the odds of reporting neglected care were lower among women that governmental employ compared to those women with Housewife (AOR: 0.24, 95%, CI (0.07–0.78)) (Table 4).

In-depth interview data shows that women who stay at the health facilities (as opposed to going home after birth) complained of neglected care due to their stay in the health facility, such as experiences of disrespect and abuse. Indeed, the main reason for their stay was that the health profession neglected care led to complications developing – akin to double disadvantage whereby they had to stay due to neglect and then suffered neglect during their stay.

## Discussion

This study aimed to assess the magnitude and factors associated with neglected and non-consented care during childbirth by using both quantitative and qualitative methods in central Tigray public hospitals. The current finding showed that the prevalence of neglected care among women giving birth was 82%. Also supported by the qualitative study in the present study. This finding is higher than in different studies conducted in western Ethiopia 25.2% [21], Kenya 14.3% [22], Ghana 31.9% [23], Sub-Saharan Africa 16.86% [24], and southwest Nigeria 37.7% [25]. This difference may be due to the difference in definition.

Most women in the present study reported that there was a lack of regular assessment during labor. The in-depth interview in the present study, as also reported in other studies in Tigray [26] and Ghana [23], corroborated that women gave birth at a health facility without the help of a health professional.

Several variables predicted the magnitude of neglected care in our setting. The odds of neglected care among women who do not receive ANC for a recent child were 8 times more likely as compared to those women who receive ANC for the recent child. This finding is consistent with other studies in Ethiopia Bale and Bahridar town [6, 27]. Participants' reporting neglected care was lower among women that governmental employ compared to those women’s that Housewife. This finding is supported by finding from Western Ethiopia [21]. This is because government employees' women are more aware of their rights, and are more likely to report any abuse. This may be explained by government employees being more aware of their rights, more sensitive to mistreatment, and very likely to report neglected care. This implies that initiatives to empower women and help them understand their rights when visiting health facilities to seek care should target women who are less educated and not formally employed.

In this study, four out of five women experienced non consented care. This finding was in line with a study conducted in Pakistan (78%) [28]. But this study was found higher than from studies conducted in Bahridar 57.6% [29], western Ethiopia 54.1% [22], Kenya 4.3% [21], southwest Nigeria 47.7% [25], and Gahanna 54.5% [7]. This discrepancy may be due to: (i) health care providers lack understanding of women's rights [30], (ii) they may not consider these non-consented care as major contributors to mistreatment [31], or (iii) these behaviors may have become normalized [32, 33]. In-depth interview results in the present study also indicated that some health professionals do not accept the consent for any routine care procedure.

Some health care professionals may practice non-consented care for the safety of women and fetuses during childbirth, but the way they communicate may be viewed as abuse by the women.

The odds of reporting non-consented care were lower among women with no formal education or completed only primary education, compared to those more educated women. This finding is consistent with other studies in Ethiopia [34] and Tanzanian [35]. This is because highly educated women are more aware of their rights, are more likely to report any abuse, and have a higher quality of care and a greater ability to report abuse. This implied that the Ethiopian Government and the Ministry of Health need to focus education on women's empowerment and educate women so that they can understand their rights to service provision at health facilities.

Women who had an episiotomy or tear during delivery were four times more likely to non-consented care compared to women who had an instrumental delivery. These manifestations may be more common because service providers lack an understanding of women's rights [30] and workloads for the implementation of consent for each practice.

The odds of non-consented care among male than female health workers was low. This finding is consistent with other studies in Jimma University, Southwest Ethiopia [34]. A finding which needs further investigation. A literature review on barriers to quality midwifery care discussed the triple burdens faced by female midwives: (1) reproductive (childbearing), (2) productive (economic), and (3) community management (e.g. unpaid work in support of the community). The effect of social, economic, and professional barriers resulted in moral distress and burnout, which may have led to abusive behavior [36]. The odds of non-consented care among women who had only been delivered in a health facility once was two times higher than those of women who had been delivered in a health facility twice or more. This is in agreement with the study conducted in western Ethiopia [21]. This may be because women delivered more than two times in the health facility may be familiar with the likelihood of experiencing non-consented care as compared to those primigravida women.

Although the study comprehensively addresses multiple outcomes, there are some limitations. First, it only addresses the perspectives of clients it does not provide the perspectives of health care providers. Second, the study does not include observation as a method of validation for the abuse or disrespect of women. Third, the framework used in this study was based on the landscape analysis developed by Bowser and Hill which has not yet been tested or validated and focuses specifically on facility-based childbirth more recently, experts have broadened the scope of neglected and non-consented care by incorporating systematic failures at the health system level that may directly or indirectly (via service providers) lead to neglected and non-consented care [15, 20].

## Conclusions

This study reveals the substantial extent of non-consented and non-confidential care during delivery and the urgent need for intervention in this region of East Africa. The educational level of the women, mode of recent deliveries, Sex of HCP conducting delivery, number of deliveries in a Health Centre, and receiving ANC for the recent child were risk factors with neglected and non-consented care. This shows that mistreatment is a critical problem in the study area and it needs urgent intervention including strengthening of awareness of both patients and healthcare providers on patients' rights and responsibilities and training service providers in patient-centered care and interpersonal communication and relationships to minimize mistreatment.

## Data Availability

The dataset supporting the conclusions of this article is included in the article.

## Abbreviations

ANC: Antenatal Care
CRC: Compassionate And Respectful care
EDHS: Ethiopian Demographic Health Survey
HCP: Health Care Provider
HIV/AIDS: Human Immune Deficiency Virus/Acquired Immune Deficiency
HSTP: Health Sector Transformation Agenda Plan
LD: Labor and Delivery
MMR: Maternal Mortality Ratio
MMR: Maternal Mortality Rate
MOH: Ministry Of Health
NICU: Neonatal Intensive Care Unit
NGO: Nongovernmental Organization
PN: Post Natal Care
RMC: Respectful Maternity Care
SDG: Sustainable Development Goal
TRA: Translating Research to Action
WHO: World Health Organization

## References

[1] Belali-Oskuei S (2019). Prenatal care utilization of pregnant women in East Azarbaijan Province. Depiction of Health.

[2] Ministry of Health (2018). Reproductive, maternal, neonatal, and child health program overview and pharmaceuticals management training for pharmacy professionals. Participant Manual.

[3] LP F, ME K. Disrespect and abuse of women in childbirth: challenging the global quality and accountability agendas. Lancet. 2014;384(9948):e42-4. 10.1016/S0140-6736(14)60859-X 24965825

[4] Pitter CP, et al. Disrespectful maternity care: a threat to the maternal health 2030 Agenda in Jamaica. Int J Women’s Health Well. 2017;3(057):1353-2474.

[5] 2011, E.E.D.a.H.S., Ethiopia Demographic and Health Survey Central Statistical Agency Addis Ababa, Ethiopia Calverton, Maryland, USA ICF (International Classification of Functioning, Disability, and Health), 2011.

[6] Mekonnen A, Fikadu G, Esmeal A (2019). Disrespectful and abusive maternity care during childbirth in Bale zone Public Hospitals Southeast Ethiopia: cross-sectional study. Clin Pract.

[7] Wassihun B (2018). Prevalence of disrespect and abuse of women during childbirth and associated factors in Bahir Dar town Ethiopia. Epidemiol Health.

[8] TekleBobo F (2019). Disrespect and abuse during childbirth in Western Ethiopia: should women continue to tolerate?. PloS One.

[9] Wassihun B, Zeleke S (2018). Compassionate and respectful maternity care during facility-based childbirth and women's intent to use maternity service in Bahir Dar, Ethiopia. BMC Pregnancy Childbirth.

[10] Sheferaw ED (2017). Respectful maternity care in Ethiopian public health facilities. Reprod Health.

[11] Bobo FT (2019). Disrespect and abuse during childbirth in Western Ethiopia: Should women continue to tolerate?. PloS One.

[12] Okonofua F (2017). Qualitative assessment of women’s satisfaction with maternal health care in referral hospitals in Nigeria. Reprod Health.

[13] World Health Organization (2014). Trends in maternal mortality: 1990 to 2013: in estimates by WHO, UNICEF, UNFPA, the World Bank and the United Nations Population Division.

[14] Wassihun B, Zeleke S (2018). Compassionate and respectful maternity care during facility-based childbirth and womenâ€™s intent to use maternity service in Bahir Dar, Ethiopia. BMC Pregnancy Childbirth.

[15] Bowser D, Hill K (2010). Exploring evidence for disrespect and abuse in facility-based childbirth.

[16] World Health Organization and UNICEF (2014). Trends in maternal mortality: 1990 to 2013: estimates by WHO.

[17] Sommer M (2003). National labor law profile: Federal democratic republic of Ethiopia.

[18] Pearson L (2011). User fees, and maternity services in Ethiopia. Int J Gynecol Obstet.

[19] Reis V (2016). Respectful maternity care. Country experience.

[20] Bowser D, Hill K (2010). Exploring evidence for disrespect and abuse in facility-based childbirth.

[21] Bobo FT (2019). Disrespect and abuse during childbirth in Western Ethiopia: should women continue to tolerate?. PloS One.

[22] Abuya T (2015). Exploring the prevalence of disrespect and abuse during childbirth in Kenya. PloS One.

[23] Ganle JK, Krampah E. Mistreatment of women in health facilities by midwives during childbirth in Ghana: prevalence and associated factors, in Selected Topics in Midwifery Care. IntechOpen, Open Access books; 2018. 10.5772/intechopen.82432.

[24] Kassa ZY, Tsegaye B, Abeje A (2020). Disrespect and abuse of women during the process of childbirth at health facilities in sub-Saharan Africa: a systematic review and meta-analysis. BMC Int Health Hum Rights.

[25] Ogunlaja A (2017). Respectful maternity care" or "disrespect and abuse during maternity care"; experience of pregnant women in Ogbomosho, southwest Nigeria. Rwanda Med J.

[26] Gebremichael MW (2018). Women suffer more from disrespectful and abusive care than from the labor pain itself: a qualitative study from Women's perspective. BMC Pregnancy Childbirth.

[27] Wassihun B, Zeleke S (2018). Compassionate and respectful maternity care during facility-based childbirth and women's intent to use maternity service in Bahir Dar Ethiopia. BMC Pregnancy Childbirth.

[28] Hameed W, Avan BI (2018). Women's experiences of mistreatment during childbirth: a comparative view of home-and facility-based births in Pakistan. PloS One.

[29] Okafor II, Ugwu EO, Obi SN (2015). Disrespect and abuse during facility-based childbirth in a low-income country. Int J Gynecol Obstet.

[30] Kruske S (2013). Maternity care providers’ perceptions of women’s autonomy and the law. BMC Pregnancy Childbirth.

[31] Balde MD (2017). Perceptions and experiences of the mistreatment of women during childbirth in health facilities in Guinea: a qualitative study with women and service providers. Reprod Health.

[32] Bohren MA (2016). “By slapping their laps, the patient will know that you truly care for her”: a qualitative study on social norms and acceptability of the mistreatment of women during childbirth in Abuja Nigeria. SSM-Popul Health.

[33] Freedman LP (2014). Defining disrespect and abuse of women in childbirth: a research, policy and rights agenda. Bull World Health Organ.

[34] Siraj A, Teka W, Hebo H (2019). Prevalence of disrespect and abuse during facility-based childbirth and associated factors, Jimma University Medical Center Southwest Ethiopia. BMC Pregnancy Childbirth.

[35] Kruk ME (2018). Disrespectful and abusive treatment during facility delivery in Tanzania: a facility and community survey. Health Policy Plan.

[36] Filby A, McConville F, Portela A (2016). What prevents quality midwifery care? A systematic mapping of barriers in low and middle-income countries from the provider perspective. PloS one.

